# Effect of *TP53* contact and conformational mutations on cell survival and erythropoiesis of human hematopoietic stem cells in a long term culture model

**DOI:** 10.18632/oncotarget.25581

**Published:** 2018-07-06

**Authors:** Azam Salari, Kathrin Thomay, Jana Lentes, Juliane Ebersold, Maike Hagedorn, Britta Skawran, Claudia Davenport, Axel Schambach, Brigitte Schlegelberger, Gudrun Göhring

**Affiliations:** ^1^ Department of Human Genetics, Hannover Medical School, 30625 Hannover, Germany; ^2^ Institute of Experimental Hematology, Hannover Medical School, 30625 Hannover, Germany

**Keywords:** TP53 mutation, hematopoietic stem cells (HSCs), haematopoiesis, erythropoiesis, MDS

## Abstract

*TP53* deficiencies characterize myeloid malignancies with a dismal prognosis. To unravel the pathomechanism of *TP53* mutations in the development of myeloid malignancies, we analyzed the functional properties of *TP53* conformational and contact mutations and *TP53* loss in human CD34+ cells. We show for the first time that the analyzed conformational mutations lead to higher cell viability in human hematopoietic stem progenitor cells. In contrast to these conformational mutations, contact mutations interfered with efficient erythropoiesis.

These findings show that not only the detection of a *TP53* mutation is important, but also the specific mutation may play a role in malignant transformation and progression.

## INTRODUCTION

In myelodysplastic syndrome (MDS) and acute myeloid leukemia (AML), *TP53* mutation and *TP53* loss is associated with clonal evolution, complex karyotypes and a very poor prognosis [1, 2]. Notably, small *TP53* mutated clones predict lenalidomide resistance and disease progression in patients with del(5q) MDS [3, 4]. The majority of *TP53* mutations observed in human cancers abrogate their ability to bind and activate wt*TP*53 target genes due to loss of function (LOF). Furthermore, mutant *TP53* may exhibit dominant-negative effects (DNE) through hetero-oligomerization with the wtTP53 protein. However, some mutations not only cause the loss of the tumor suppressor ability and acquire a DNE, but also introduce oncogenic properties, which are known as gain of function (GOF) that can be detected in *TP53* null status [5]. In over half of human cancers *TP53* is mutated. The majority of these mutations are missense mutations located in the DNA-binding domain of the TP53 protein. Based on their structural and functional properties, *TP53* mutations are classified into two categories, DNA contact mutations including mutations in residues directly involved in DNA binding such as R248W and R273H and conformational mutations, which dramatically change the TP53 conformation such as R175H and R249S. The four mentioned *TP53* hotspot mutations, R175H, R248W, R249S and R273H, have been reported recently to occur in myeloid malignancies (reviewed in Table 1). Characterization of *TP53* mutations and their related pathogenesis has led to diverse and in some cases contradictory outcomes [6, 7], mainly caused by cellular context differences, which may modulate TP53 function [8]. The specific impact of *TP53* alterations and mutations on hematopoietic stem progenitor cell (HSPC) function and hematopoiesis is still poorly understood and the available information has been primarily obtained from mouse models [9]. Unravelling the pathomechanism of *TP53* mutations in the development of myeloid malignancies will, therefore, increase our knowledge on the role of specific *TP53* mutations in malignant transformation and may facilitate identification of their prognostic relevance.

**Table 1:** Prevalence of TP53 hotspot mutations in Myelodysplastic syndromes (MDS) and Acute myeloid leukemia (AML)

*TP53* Mutation	Frequency in AML/MDS [%]			
	IARC^1^	Papaemmanuil *et al*., 2016^2^	TP53 Handbook^3^	Ohgami *et al*., 2015^4^
R248W	1.62	2.04	6.03	1.10
R175H	4.76	7.14	2.59	3.30
R273H	2.70	3.06	4.30	0.00
R249S	0.22	0.00	n/a	0.00

^1^IARC *p53* mutation database.

^2^Papaemmanuil E, Gerstung M, Bullinger L, Gaidzik VI, Paschka P, Roberts ND, Potter NE, Heuser M, Thol F, Bolli N, Gundem G, Van Loo P, Martincorena I, et al. Genomic Classification and Prognosis in Acute Myeloid Leukemia. The New England Journal of Medicine. 2016; 374:2209–21.

^3^Hjortsberg L, Rubio-Nevado JM, Hamroun D, Béroud C, Claustre M, Soussi T. The p53 Mutation handbook 2.0, available online; http://p53.free.fr.

^4^Ohgami RS, Ma L, Merker JD, Gotlib JR, Schrijver I, Zehnder JL, Arber DA. Next-generation sequencing of acute myeloid leukemia identifies the significance of TP53, U2AF1, ASXL1, and TET2 mutations. Modern Pathology. 2015; 28:706–14.

## RESULTS

### Accumulation of *TP53* mutations in CD34+ cells does not result in activation of *TP53* target genes

**Figure 1:**
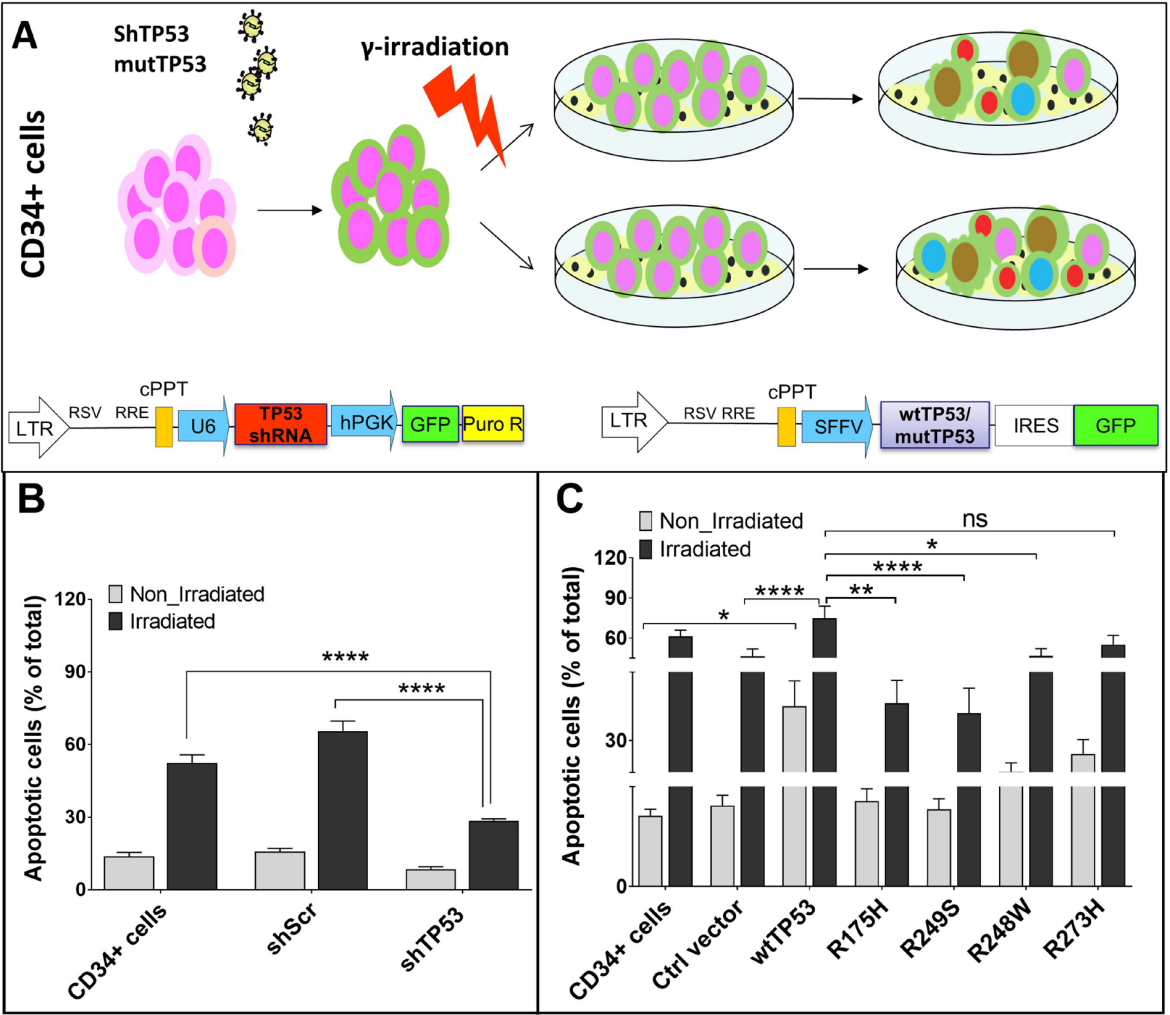
Vector constructs and their influence on apoptosis of CD34+ cells (**A**) Overview of the workflow. (**B**) Under stress conditions apoptosis rate of CD34+ cells transduced with shTP53 is significantly lower than in CD34+ cells transduced with shScr or in non-transduced cells. (**C**) Under stress conditions CD34+ cells over-expressing wtTP53 have significantly higher apoptosis rates in comparison to controls and to cells with *TP53* mutations R175H, R248W and R249S. (^*^P < 0.05; ^**^P < 0.01; ^***^P < 0.001).

To better understand the pathomechanism of *TP53* mutations in the development of myeloid malignancies, we analyzed the functional properties of the different *TP53* mutations or *TP53* loss in human cord blood HSPCs from healthy donors. For this purpose, lentiviral constructs were designed to overexpress either wt*TP53* or one of the hotspot mutations R175H, R248W, R249S and R273H. Human cord blood CD34+ cells were then transduced with viral supernatants to reflect the occurrence of complex clones in myeloid malignancies. In addition, *TP53* was downregulated by a shRNA construct (shTP53). After transduction, cells were sorted and half of these cells were irradiated (γ-radiation, 2 Gy) and maintained in long-term culture on irradiated feeder cells for 6 weeks. Irradiation was performed as an additional stressor of the cells in order to better understand the influence of *TP53* alterations. Functional assays were performed 48 hours post-sorting, after 3 and 6 weeks (Figure 1A, Supplementary Figure 1).

Quantitative real-time PCR (RT-qPCR) was used to determine the influence of *TP53* mutation status on mRNA stability. The four TP53 mutants showed increased mRNA levels when compared to the wild type control (Supplementary Figure 2A). In order to determine the influence that the different *TP53* mutations may have on the TP53 function, the expression of TP53 target genes including BAX, p21 (CDKN1A) and MDM2 was measured by RT-qPCR in transduced CD34+ cells in comparison to the non-transduced control cells. Only the over-expression of wild type TP53 induced the expression of TP53 target genes, whereas the overexpression of the four TP53 mutants did not result in activation of TP53 target genes (Supplementary Figure 2B). Additionally the protein levels were measured by Western Blot analyses and represented the mRNA expression (Supplementary Figure 3).

### Presence of TP53 conformational mutations improves the cell survival

Functional TP53 can induce apoptosis in damaged cells. Accordingly, we found dramatically lower apoptosis rate for the irradiated cells transduced with shTP53 compared to the controls (Figure 1B). We further investigated the effect of overexpression of wt*TP53* and the four hotspot *TP53* mutations on apoptosis under normal and stress conditions. Here, we found significantly higher apoptosis rates in CD34+ cells transduced with wt*TP53* in comparison to the control vector and the four *TP53* hotspot mutations. The apoptosis rate further increased after irradiation. Interestingly, CD34+ cells transduced with contact mutations (R248W and R273H) showed higher apoptosis rates than those transduced with conformational mutations (R175H and R249S). This indicates a differential impact of *TP53* contact and conformational mutations on apoptosis (Figure 1C).

### Conformational mutations R175H and R249S prevent the impairment of hemato- and erythropoiesis induced by endogenous wildtype TP53

**Figure 2:**
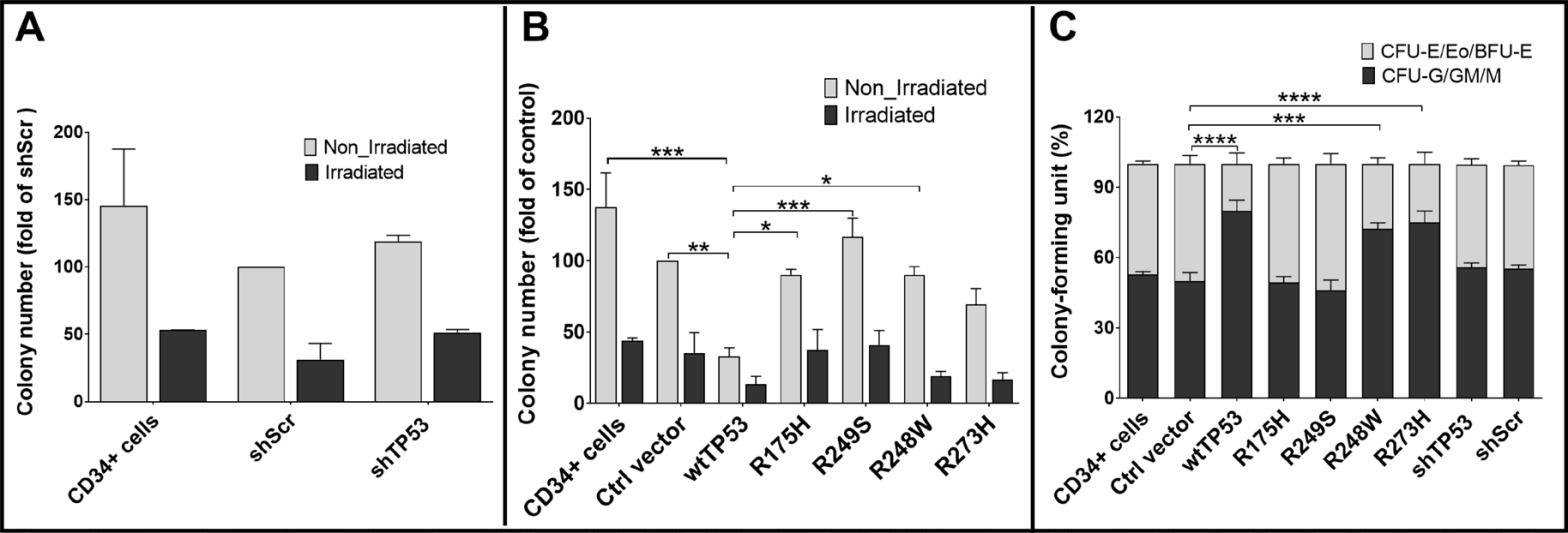
Influence of TP53 hotspot mutations on hematopoiesis in colony forming unit (CFU) assays (**A**) CFU results of knockdown of TP53 by shRNA: knockdown of TP53 did not influence colony numbers of CD34+ cells as compared to shScr as a control. (**B**) CFU results of knockdown of cells transduced with wt*TP53* or *TP53* mutations. After irradiation there was a weaker reduction in cell colony numbers in CD34+ cells transduced with conformational mutations (R175H and R249S), whereas CD34+ cells transduced with contact mutations (R248W and R273H) did not differ from those transduced with wt*TP53.* (**C**) Erythropoiesis in CD34+ cells overexpressing wtTP53 or expressing the contact mutations R248W and R273H was significantly reduced as compared to CD34+ cells transduced with conformational mutations. (^*^P < 0.05; ^**^P < 0.01; ^***^P < 0.001).

We performed colony forming unit assays (CFU) to further compare the differential effects of contact and conformational TP53 modifications on hematopoiesis. While irradiation was found to strongly impair hematopoiesis, knockdown of TP53 did not influence colony numbers of CD34+ cells as compared to the control shScr (Figure 2A). In both normal and irradiated CD34+ cells, transduction with wt*TP53* was associated with a significant reduction in the number of colonies. However, the effect of transduction with the conformational (R175H and R249S) or contact (R248W and R273H) mutations on colony forming capacity was differentially regulated under stress conditions. While none of the hot spot mutations could strongly alter the number of colonies in the non-irradiated cells, irradiation of cells transduced with contact mutations showed a significant reduction of colony numbers, comparable to those observed in wt*TP53*. In contrast, irradiation of transduced cells with conformational mutants had no effect on colony numbers (Figure 2B). In addition, erythropoiesis in CD34+ cells overexpressing wt*TP53* or expressing the contact mutations was significantly reduced in comparison to those transduced with conformational mutations (R175H and R249S, Figure 2C, Supplementary Figure 4).

The differences were also confirmed by CD71 staining. CD71 or the transferrin receptor is highly expressed on the surface of cells of the erythroid lineage. In accordance with the previous results, the ratio of CD71+ cells overexpressing wt*TP53* or the contact mutations was lower in comparison to those transduced with conformational mutations or the control vector (Supplementary Figure 5). A comparable effect was detected for irradiated cells. Thus, only the conformational mutations R175H and R249S prevented the impairment of hemato- and erythropoiesis induced by endogenous wildtype TP53.

### TP53 dysfunction is associated with chromosomal instability in CD34+ cells upon exposure to irradiation

Efficiency of TP53 function in protecting the genome stability was assessed by karyotyping and also by measuring the γH2AX foci indicating DNA double strand break (DSB) during the long term culture, at the end of weeks 1, 3 and 6. Under non-irradiated conditions no increased chromosomal stability was detected in transduced CD34+ cells. However, upon the exposure to cellular stress by gama-irradiation (2 Gy) chromosomal instability was observed in CD34+ cells with p53 deficiency (Supplementary Figure 6A and 6B). Measuring the efficiency of DSB repair by γH2AX foci showed higher DSB damage in irradiated cells compared to the non-irradiated ones with no discernible difference among cells expressing different TP53 mutants (Supplementary Figure 6C and 6D). Thus, the expression of a *TP53* mutation by itself is not sufficient to introduce chromosomal instability. A second hit, in our case the irradiation, is needed to result in chromosomal instability.

## DISCUSSION

*TP53* mutations are considered as an independent prognostic indicator of response to chemotherapy and survival in myeloid malignancies [10, 11]. In this study TP53 status of HSPC CD34+ cells was modified and functional properties of the modified cells during hematopoiesis were assessed. While wild type TP53 induced expression of its target genes, overexpressed TP53 mutants failed to induce the expression of TP53 target genes including BAX, p21 (CDKN1A) and MDM2.

To better understand the effect of *TP53* mutation and loss, we performed lentiviral transductions of human cord blood CD34+ cells from healthy donors, using lentiviral shRNA targeting TP53 for its downregulation. Furthermore, we performed lentiviral transduction of wt*TP53* and four hotspot mutations (R175H, R248W, R249S, R273H).

In the hematopoietic system p53 target genes involved in apoptotic induction predominantly determine cell fate [12]. According to our data *TP53* modifications induce apoptosis differentially. Albeit the differences were not significant, *TP53* contact mutations (R248W and R273H) induced higher rates of apoptosis in CD34+ cells than conformational mutations (R175H and R249S). This resulted in lower survival rates and in lower numbers of hematopoietic colonies. Under stress conditions, impairment of apoptosis induction and proliferation of hematopoietic cells was also stronger for conformational mutations (R175H and R249S) than for contact mutations. HSPCs have the ability to differentiate into all blood cell lineages and to self-renew [13]. *TP53* influences this capability by regulating self-renewal and quiescence of HSCs [14]. In *TP53* knockout mice hematopoiesis is overactivated leading to higher cellular proliferation rate to increase the HSC pool [9]. In this human long term culture model we did not observe a reduction of colony numbers after TP53 knockdown. Yet our results provide evidence that in addition to wt*TP53*, accumulation of *TP*53 contact mutations (R248W and R273H) impair the erythroid differentiation potential of HSPCs. We showed that expression of TP53 contact mutations also interferes with efficient erythropoiesis. TP53 conformational mutations (R175H and R249S) interfere with apoptosis induction by binding to members of the TP53 family and thereby restricting their function, which might be the reason for the observed differences in erythropoiesis between contact and conformational mutations, mediated by R175H and R249S, respectively. Previous reports on enhancing proliferation and resistance to apoptosis derived by conformational mutations, especially R175H, and increased apoptosis deduced by contact mutations, especially R273H, are in agreement with our findings [15–17].

TP53 target genes have previously been reported to be involved in determining the hematopoietic stem cell fate [16, 18]. By binding to TP53 family members, conformationally mutated TP53 may restrict their function and, thereby, interfere with apoptosis [19, 20]. Cooperation between wtTP53 and its family members is essential for apoptosis induction [21]. Apoptosis induced by overexpression of wtTP53 or TP53 contact mutations is highly restricted to the erythroid lineages and may affect their efficient differentiation. A similar phenotype has been reported for diseases with deficient ribosomal biogenesis such as Diamond-Blackfan anemia (DBA), Dyskeratosis congenita (DKC) and del5q MDS where inactivation of MDM2 results to TP53 accumulation [22–26]. Our data show the potential destructive role of overactivated wild type TP53 or its contact mutations, Recently, a case of Fanconi anemia (FA) has been reported with hyper-activation of TP53/p21 (CDKN1A) pathway as the major cause of bone marrow failure [27]. In line with our results, the accumulation and activation of TP53 as a result of MDM2 inactivation has been reported as a possible mechanism behind defected ribosome biogenesis as seen in DBA, DKC and del5q MDS [16, 18, 28]. However, the specific role of TP53 remains to be elucidated. We show that the presence of TP53 contact mutations may have the same effect as wtTP53 activation, indicating a possible involvement in these diseases. The observed reduction in erythropoiesis as observed may result from an inhibition of GATA1, an erythroid-lineage specific transcription factor which protects the erythroid differentiation and survival [21, 29–32].

In this study, we show for the first time that only specific conformational mutations (R175H and R249S) lead to higher cell viability in human HSPC and that only these mutations can interfere with efficient erythropoiesis. These findings show that not only the detection of a *TP53* mutation is important, but also the specific mutation may play a role in malignant transformation. It still remains unclear if inactivation of TP53 directly increases chromosomal instability, if it allows chromosomally unstable hematopoietic stem cells to bypass senescence or apoptosis and survive, and if and how this is mechanistically implicated in the development of clones with complex karyotypes in MDS.

## MATERIALS AND METHODS

### Vector design

In order to downregulate *TP53*, a new lentivirus construct was generated by replacing the puro cassette of shRNATP53 PLKO.1 vector (addgene #19119) with the GFP-Puro cassette of pRRL.PPT.SF.GFPpuro.Pre vector, the GFP-Puro Cassette was also inserted in to the scramble shRNA vector (addgene #1864), which was used as a control.

In order to overexpress wtTP53 or its mutations, a new lentivirus construct was generated by replacing the wtTP53 cassette from pCMV-Neo-Bam p53 wt vector (addgene #16434) with the mCherry cassette from pRRL.PPT.SF.mcherry iGFP pre vector. Then, four missense mutations of *TP53* (R175H, R248W, R249S, R273H) were separately introduced in to the pRRL.PPT.SF.wt/mutp53 iGFP.pre vector by PCR mutagenesis and the results were confirmed by Sanger sequencing (GATC Biotech). The empty vector was used as control.

### Cell preparation and CD34^+^ cell purification

CD34+ cells were isolated from umbilical cord blood (CB) of healthy donors. Mononuclear cells (MNCs) were isolated from CB by density gradient centrifugation using Ficoll-Paque PLUS (GE Healthcare, Sweden). The CD34+ cell fraction was then isolated from the MNCs using the Midi MACS system (CD34+ Microbead Kit; Miltenyi Biotec; Bergisch Gladbach, Germany) according to the manufacturer's instructions. Briefly, the MNCs were washed, resuspended in PBS containing 0.5% BSA (Santa Cruz Biotechnology, USA) and 2 mM EDTA, and subjected to an incubation step with 150 μl FcR blocking reagent provided by the manufacturer (Miltenyi Biotec) and 150 μl CD34 microbeads with up to 1 × 10^8^ total cells for 30 min at 4°C. Labeled cells were separated by loading the cells in to the column in a magnetic field. Trapped cells were eluted after removal of the column from the magnet and CD34+ cell purity was enriched up to 95% by loading the eluted fraction over a second magnetic column. Study of CD34+ cells was approved by the ethics committee of Hannover Medical School (No. 835–2010). Umbilical cord blood was donated after written informed consent by women after child birth at Hannover Medical School.

### Production of lentiviruses

Third-generation “self-inactivating” (SIN) high titer lentiviruses were generated by transient co-transfection of 293T cells of four plasmids as follows:

1 × 10^7^ 293T cells in a 10 cm culture dish were transfected using standard calcium phosphate precipitation (Calcium Phosphate Transfection Kit, Sigma-Aldrich), according to the manufacturer's instructions, with 5 μg shp53 or *TP53* mutations, 10 μg pMDLg/pRRE (addgene #12251), 5 μg pRSV-Rev (addgene #12253) and 2 μg RD114/TR plasmids. Supernatants containing the viral particles were collected every 12 hours between 16–72 h after transfection, filtered through a 0.22 μm filter and frozen at −80°C. All viral supernatants were thawed, combined and concentrated using ultra-15 centrifugal filter units (Amicon) for up to 100× and stored in aliquots at −80°C.

### Transduction of CD34^+^ cells

CD34+ cells were cultured in Stem Span medium (Stem Cell Technologies) containing cytokines (hFlt3 100 ng/ml, hSCF 100 ng/ml, hTPO 20 ng/ml, hIL-6 20 ng/ml, Peprotech) and 1% Penicillin-Streptomycin (Biochrom) 12 h before transduction. Transduction of pre-stimulated cells was done in RetroNectin (Takara, Shiga, Japan)-coated plates according to the manufacturer's instructions. Briefly, cells were seeded in a coated 6-well plate, then concentrated viral supernatants (MOI 2.5) and protamine sulfate (4 μg/ml) were added to the cells and the plate was centrifuged for 2 h at 31°C, 1,500 × g. Cells were incubated for 12 h, then fresh medium was added to the cells and 12 h later medium was exchanged. 48 h after transduction cells were sorted for GFP^+^ cells by fluorescence-activated cell sorting (FACS).

### Long-term culture of cD34^+^ cells

Transduced CD34+ cells were co-cultured with irradiated (80 Gy) murine fibroblasts that constitutively secrete human growth factors (M2-10B4, CLS Cell Lines Service). Before irradiation, M2-10B4 cells were grown in RPMI medium (Biochrom) with 10% FCS (FBS Superior, Biochrom) and 1% Penicillin-Streptomycin (Biochrom). CD34+ cells were cultured in Stem Span medium supplied with cytokines (human Flt3-Ligand, hFlt3 100 ng/ml, human stem cell Factor, hSCF 100 ng/ml, human thrombopoietin, hTPO 20 ng/ml, human interleukin 6, hIL-6 20 ng/ml, Peprotech) and 1% Penicillin-Streptomycin (Biochrom), through the transduction and sorting of transduced cells. Transduced CD34+ cells were grown for at least 6 weeks on irradiated M2-10B4 cells according to standard procedures in MyeloCult (Stem Cell Technologies). For optimal growth hydrocor-tisone (10^−6^ M, Stem Cell Technologies) and cytokines (hFlt3 20 ng/ml, hSCF 20 ng/ml, hTPO 4 ng/ml, hIL-6 4 ng/ml, Peprotech) were added. On a weekly basis, half the medium was changed and used for analysis.

### Apoptosis assay

For Apoptosis assay, cells were co-stained with Annexin V-APC and 7-AAD (BD Pharmingen™), and analysed for apoptosis by flow cytometry using a FACSCalibur (BD Biosciences). Briefly, 4 × 10^3^ cells were washed twice with phosphate-buffered saline (PBS), resuspended in 1 × Binding Buffer (10 mM HEPES, pH 7.4, 140 mM NaCl, 2.5 mM CaCl_2_) and incubated with 2.5 μl each of Annexin V-APC antibody and 7-AAD in the dark for 15 min. Both early-apoptotic (7AAD-/Annexin V+) and late-apoptotic (7AAD+/Annexin V+) cells were included in the quantification using FlowJo software (Tree Star).

### Colony forming unit assay

Colony forming unit assay was done according to the manufacturer's instructions (MethoCult H4435; Stem Cell Technologies). Briefly, cells (1 × 10^3^ /dich) were plated in duplicate into methylcellulose in a 35 mm dish. The cells were incubated at 37°C in a humidified atmosphere with 5% CO_2_. Final evaluation of cell division and colony formation was conducted at day 14 of culture.

### Cell surface staining for CD71

Single cell suspensions were stained with fluorochrome conjugated antibody for CD71. Flow cytometry was performed by a FACSCalibur device (BD Biosciences) and data were analyzed with the FlowJo software (Tree Star).

### Statistical analysis

Data presented in this paper were collected from five independent long term cultures of CD34+ cells. Analysis of variance (ANOVA) was used followed by Sidak's or Dunnett's post hoc test for multiple comparisons to determine statistical significance in all experiments using GraphPad Prism software (GraphPad, San Diego, CA). Results are presented as means ± SEM. ^*^P < 0.05, ^**^P < 0.01, ^***^P < 0.001, determined by ANOVA.

## SUPPLEMENTARY MATERIALS FIGURES


