# Supplementary material for: Effect of TP53 contact and conformational mutations on cell survival and erythropoiesis of human hematopoietic stem cells in a long term culture model
Source: Oncotarget. 2018 Jul 6;9(52):29869–76. doi: 10.18632/oncotarget.25581 (PMC6057451; doi:10.18632/oncotarget.25581)
Supplement: Supplementary file 1 [file oncotarget-09-29869-s001.pdf]

Effect of *TP53* contact and conformational mutations on cell survival and erythropoiesis of human hematopoietic stem cells in a long term culture model

SUPPLEMENTARY MATERIALS

Western blot analysis

For Western blot analysis, equal amounts of protein-containing whole cell lysates (20 µg) were resolved on 12% SDS-PAGE mini-gels. Proteins were then electro transferred at 100 V to nitrocellulose membranes (Sigma-Aldrich) at room temperature with using the ice packs to keep it cool for 1.5 h. Then membranes were blocked with phosphate buffered saline containing 0.05% Tween 20 (TBS-T) and 2% slim fast (Slim Fast) at RT for 1 h, then, the membrane was cut to different parts according to the size of protein that was going to be detected. Membrane parts were incubated with primary antibodies diluted in TBS-T with 2% slim fast (p53, 1/5,000, GAPDH, 1/5,000, Santa Cruz Biotechnology), (p21, 1/1,000, MDM2, ½,000, BD Bioscience) overnight at 4° C. Then, the membrane was washed with TBS-T and incubated with horseradish peroxidase-conjugated secondary antibody for p53 and GAPDH (1:30,000, anti-mouse, BD Bioscience), for MDM2 (1/10,000, anti-mouse, BD Bioscience) and for p21 (1/5,000, anti-mouse, BD Bioscience) for 1 h at RT. Then, the membrane was washed with TBS-T and proteins were visualized using enhanced chemiluminescence (ECL) (Thermo Fisher Scientific) and FUJIFILM Luminescent Image Analyzer.

Gene expression analysis by real-time quantitative PCR (qRT-PCR)

Total RNA from CD34+ cells was purified using RNAeasy Mini Kit (Qiagen), and reverse transcribed using High-Capacity cDNA Reverse Transcription Kits (Applied Biosystems). Real-time PCR was performed with an ABI Step One Plus (Applied Biosystems) with SyberGreen PCR Master Mix. Primer pairs were from the QuatiTect® Primer Assay for p53, #QT00060235 and GAPDH, #QT00079247 (Qiagen). p21, BAX and MDM2 primers were designed with the following sequences:

p21 forward: 5′- TGGACCTGTCACTGTCTTGT

p21 Backward: 5′- TTCCTGTGGGCGGATTAGG

Bax forward: 5′- GTGGCAGCTGACATGTTTTC

Bax Backward: 5′- GGAGGAAGTCCAATG TCCAG

MDM2 forward: 5′- GGCAGGGGAGAGTGA TACAG

MDM2 Backward: 5′- TTGCAATGTGATGGAAG GGG

The PCR conditions were: 10 min at 95° C, 40 cycles of 15 sec at 95° C and 1 min at 60° C. The average Ct (cycle threshold) value for each gene was determined from triplicate reactions and levels of gene expressions relative to GAPDH were determined.

Cytogenetic analysis

Chromosome preparation and fluorescence R-banding were performed after 1, 3 and 6 weeks of long-term culture, as previously described [1]. Karyotypes were described according to the International System for Human Cytogenetic Nomenclature (Editor(s): Shaffer L.G. [2].

ϒH2AX measurement by immunostaining

Cells (on microscope slides) were rinsed with PBS, and then were fixed with 4% formaldehyde for 20 minutes at room temperature. Fixed cells were washed twice with PBS and permeabilized with 0.5% Triton X-100 for 5 minutes. They were then blocked with blocking solution (PBS containing 0.5% saponin, 0.5% BSA, 0.1% Triton X-100 and 0.02% sodium azide) for 30 minutes at room temperature. Cells were then stained with the mouse antibody anti-phospho-histone H2AX, serine 139, clone JBW301 (Millipore, Billerica, MA, USA) overnight at 4° C. Cells were then washed twice with blocking solution and stained with Alexa fluoro 647 goat anti-mouse IgG (Life Technologies, Carlsbad, CA, USA) for 30 minutes at 37° C. Slides were then washed twice with blocking solution, then washed shortly with PBS and incubated with Dapi for 10 minutes at room temperature. Antibodies were diluted in blocking solution to concentrations of 1:150 (γ-H2AX) and 1:1000 (AlexaFluor488). Imaging

Fluorescence microscopy was performed using a Zeiss Axioplan 2 microscope (Zeiss, Jena, Germany).

REFERENCES

1. Schlegelberger B, Metzke S, Harder S, Zühlke-Jenisch R, Zhang Y, Siebert R. Classical and Molecular Cytogenetics of Tumor Cells. In: Wegner RD. (eds) Diagnostic Cytogenetics. Springer Lab Manual. (Springer, Berlin, Heidelberg). 1999; 151–185.
2. Shaffer LG, Spokane W, McGowan-Jordan J, Schmid M (eds), Krager S. An International System for Human Cytogenetic Nomenclature. Basel. 2013.

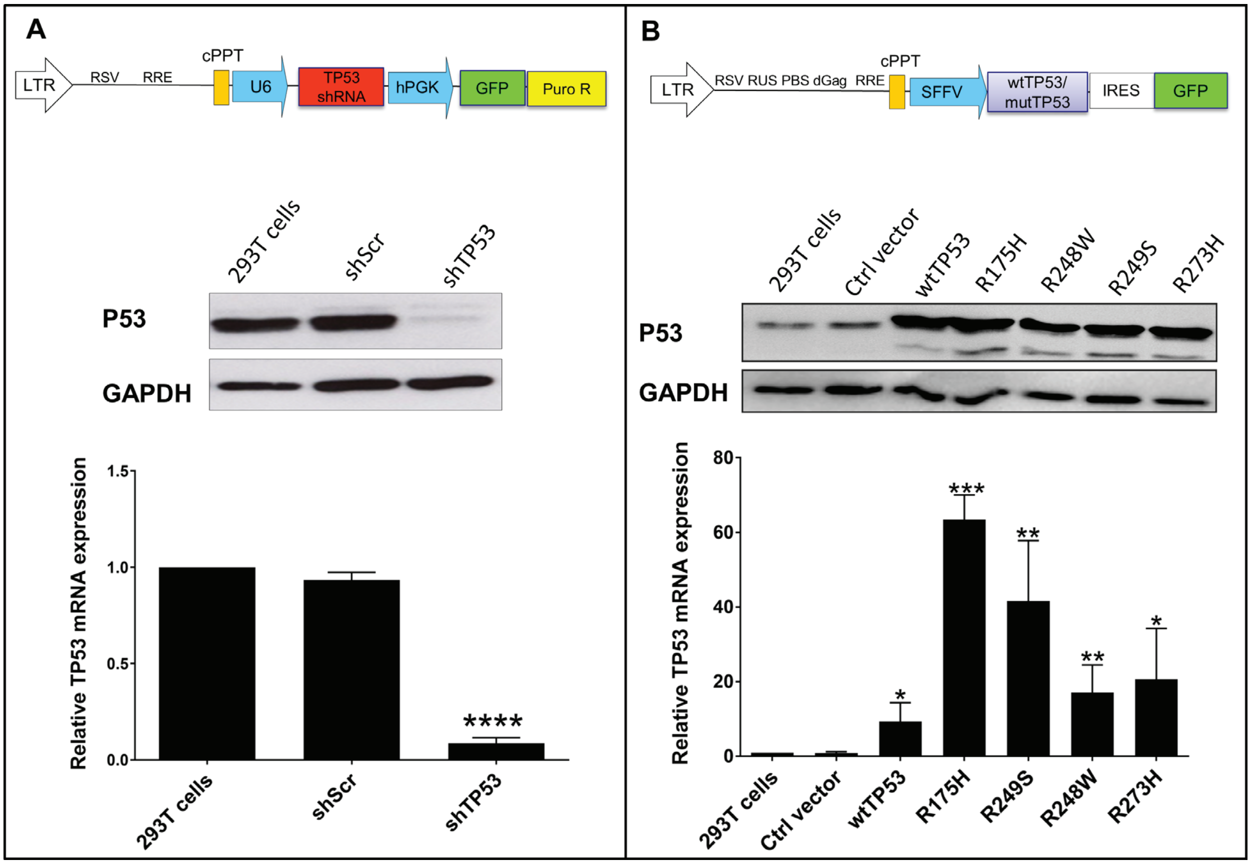

Supplementary Figure 1: Lentiviral shRNA construct and construct of *TP53* mutations lead to a strong deregulation of p53 expression. (**A**) Western blot and qRT-PCR of lentiviral shRNA contructs. (**B**) Western blot and qRT-PCR of lentiviral constructs for wt*TP53* and the four TP53 mutations. ($^{*}P < 0.05$; $^{**}P < 0.01$; $^{***}P < 0.001$).

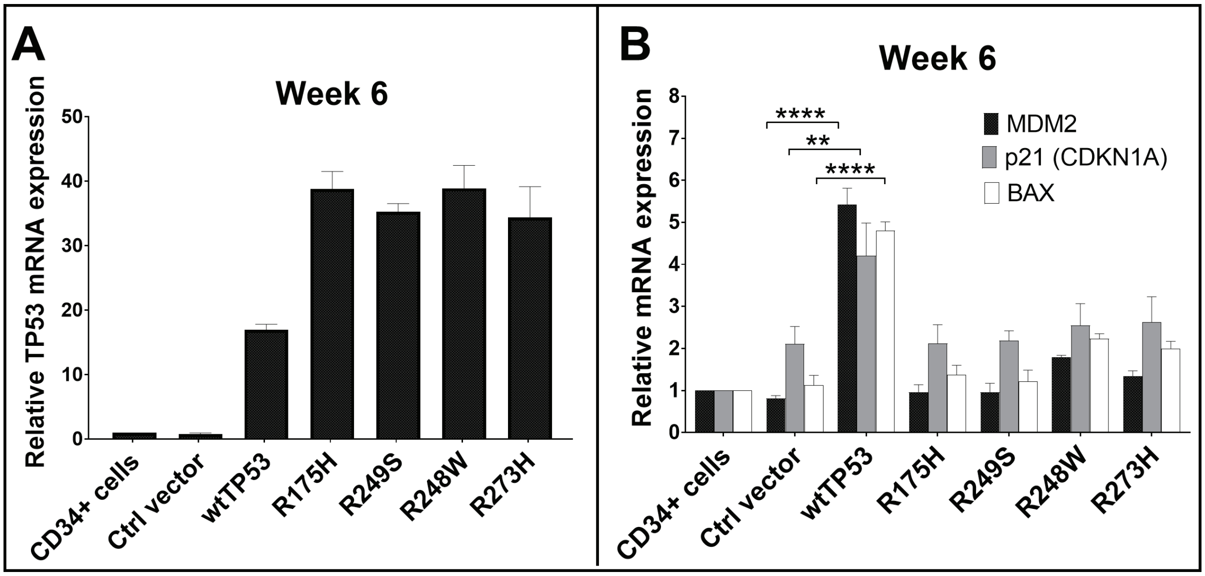

Supplementary Figure 2: Analysis of p53 mutation stability and function. Wildtype and p53 mutations were expressed in human CD34+ cells and maintained in a long term culture for six weeks. Cells were then analyzed for p53 stability and functionality at the end of culture of by measuring the expression level of p53 and its target genes by RT-qPCR. (**A**, **B**) Total RNA was isolated and expression of specific mRNAs was determined by real time quantitative PCR. (A) Analysis of p53 expression showed the stability of p53 in cells expressing p53 mutations. (B) p53 target gene analysis indicated that p53 mutations are not able to stimulate the expression of wt p53 target genes. ($^{*}P < 0.05$; $^{**}P < 0.01$; $^{***}P < 0.001$).

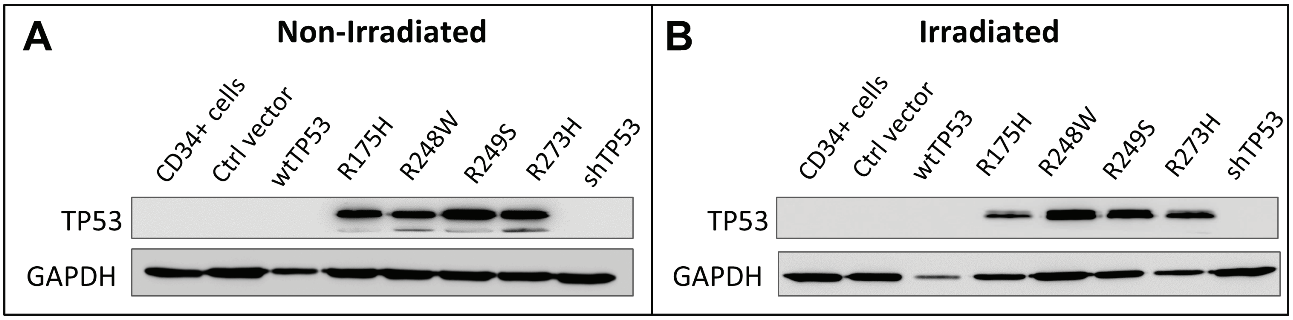

Supplementary Figure 3: Western blot analysis was done on the whole cell lysate of CD34+ cells in order to determine the abundance of p53 protein in control condition (**A**) and also after irradiation (**B**). Western blot data show that p53 mutants accumulate inside the cells.

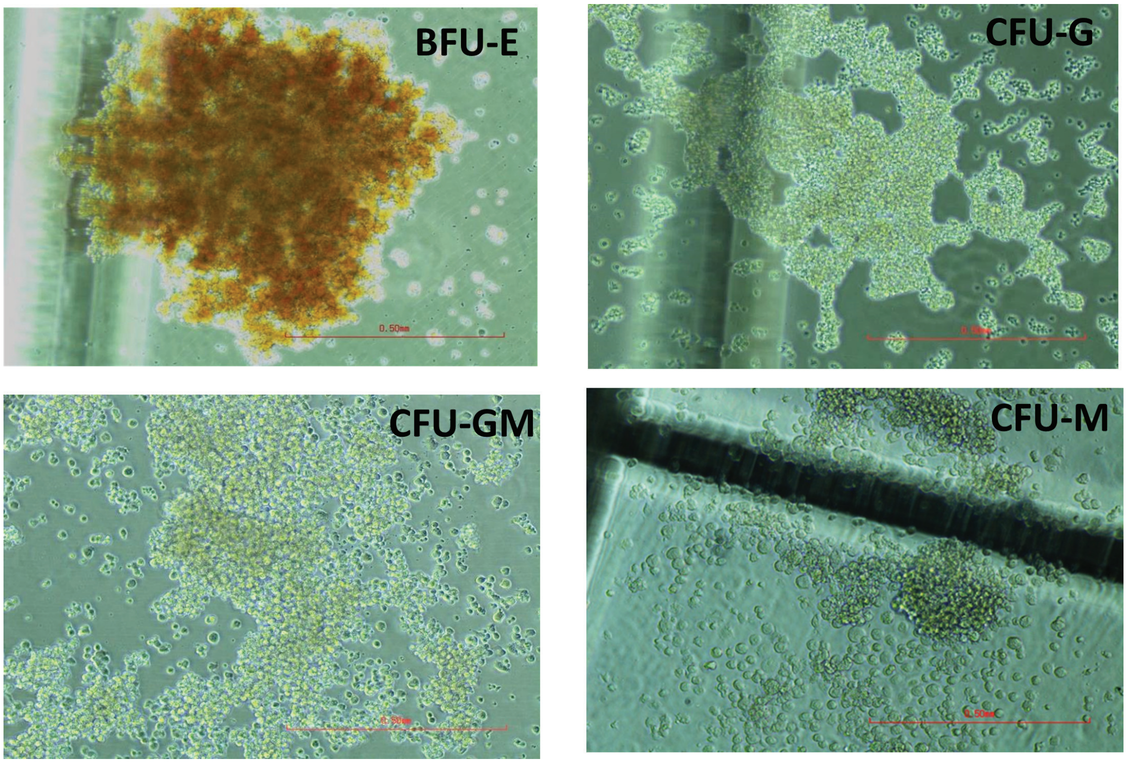

Supplementary Figure 4: CFU assay of Hematopoietic Stem Progenitor Cells (HSPCs). Colonies were observed under the inverted microscope and categorized to the erythroid or granulocytic and macrophage lineages based on their morphology and phenotypic criteria. Erythroid lineages include colony-forming unit-erythroid (CFU-E) and burst-forming unit-erythroid (BFU-E). Granulocytic and macrophage lineages include colony-forming unit – granulocyte (CFU-G), colony-forming unit – macrophage (CFU-M), colony-forming unit - granulocyte/macrophage (CFU-GM) and colony-forming unit - granulocyte/erythrocyte/macrophage/megakaryocyte CFU-GEMM.

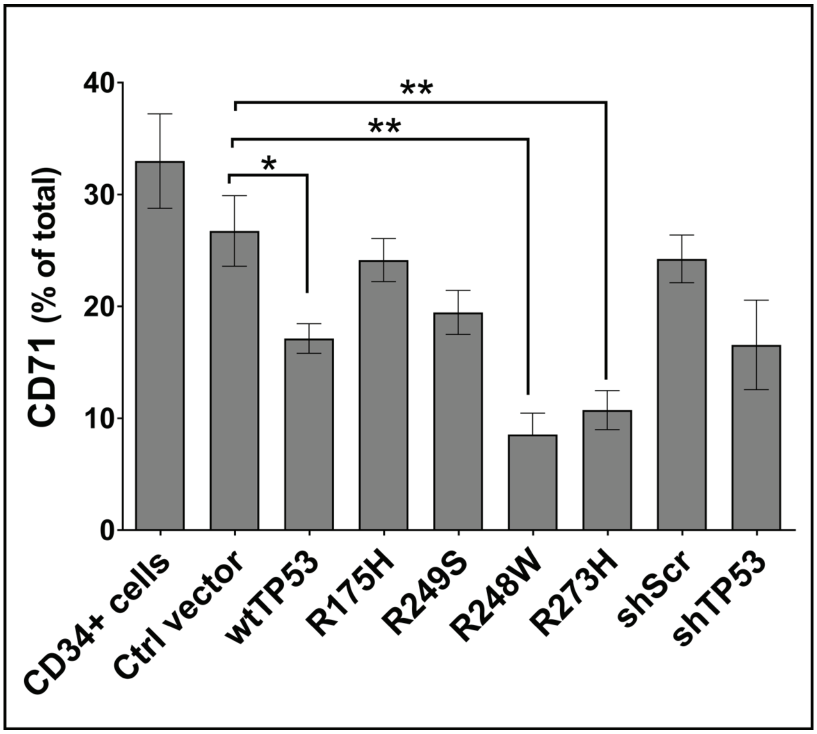

Supplementary Figure 5: Conformational mutations R175H and R249S prevent the impairment of hemato- and erythropoiesis. FACS analysis for CD71: Erythropoiesis in CD34+ cells overexpressing wtp53 or expressing the contact mutations R248W and R273H was significantly reduced as compared to CD34+ cells transduced with conformational mutations. ($^{*}P < 0.05$; $^{**}P < 0.01$).

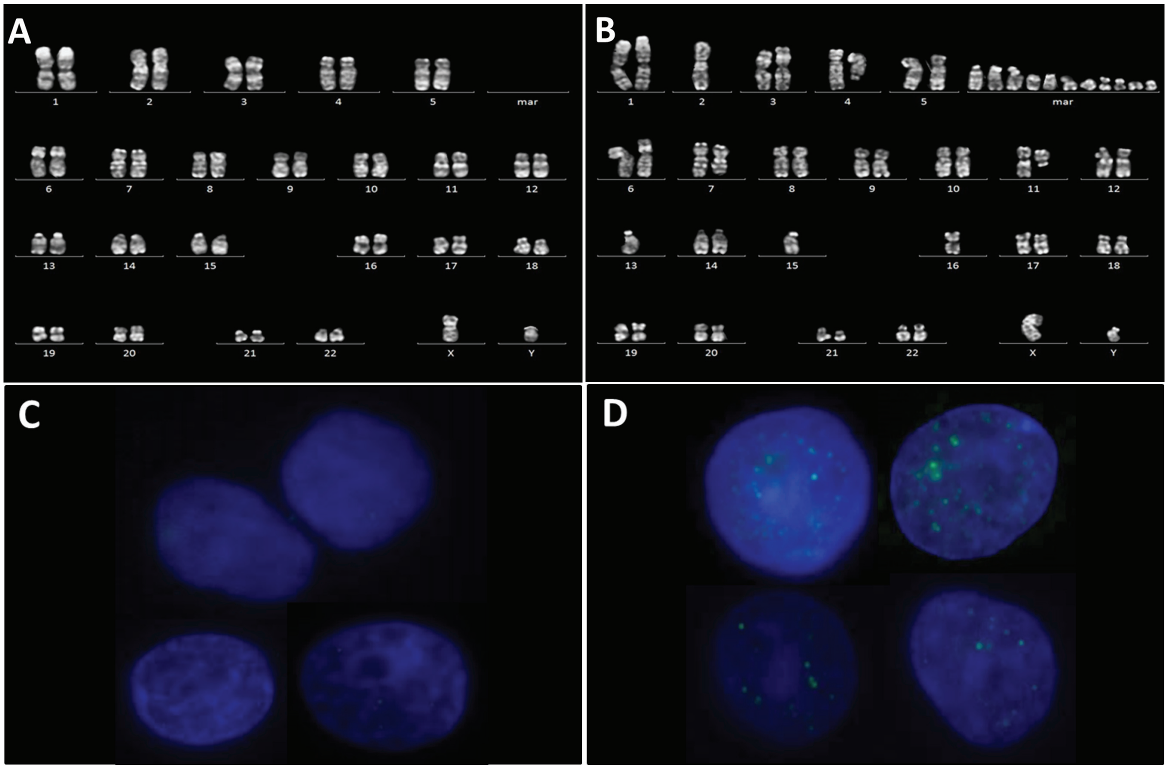

Supplementary Figure 6: p53 dysfunction is associated with chromosomal instability in CD34+ cells upon exposure to irradiation (**A**, **B**) Chromosomal stability was analyzed by fluorescence R-banding of transduced and non-transduced CD34+ cells during long term culture. (A) Karyogram of a non-irradiated, transduced CD34+ cell:no chromosomal instability is detected. (B) Karyogram of an irradiated, transduced CD34+ cell: chromosomal instability is detected. (**C**, **D**) γ-H2AX foci indicating DNA double strand breaks were measured as a marker for damage evaluation and efficiency of the DNA repair system. (C) non-irradiated, transduced CD34+ cells (D) irradiated, transduced CD34+ cells. Irradiated transduced cells showed a higher amount of foci indicating a higher amount of DNA breakages.
